# Correlation of overweight condition and obesity with mortality by COVID-19 in Brazil's state capitals

**DOI:** 10.20945/2359-3997000000351

**Published:** 2021-04-12

**Authors:** Raquel Alencastro Veiga Domingues Carneiro, Danúbia Hillesheim, Ana Luiza Curi Hallal

**Affiliations:** 1 Universidade Federal de Santa Catarina Florianópolis SC Brasil Universidade Federal de Santa Catarina, Florianópolis, SC, Brasil.

**Keywords:** Mortality, COVID-19, SARS-CoV-2, obesity, overweight

## Abstract

**Objective:**

To evaluate the correlation between the prevalence of overweight condition and obesity with mortality rates due to COVID-19 in Brazil's state capitals.

**Materials and methods:**

This is an ecological study, whose units of analysis were the 26 state capitals and the Federal District of Brazil. Prevalence was estimated by the results of the *Vigilância de Fatores de Risco e Proteção para Doenças Crônicas por Inquérito Telefônico* 2019 (VIGITEL). The general mortality rates due to COVID-19 were collected on the official website of the Brazilian Ministry of Health (MH) and stratified by the same Brazilian capitals evaluated in the VIGITEL survey. The rates included the period between the 1st and 29th Epidemiological Weeks of 2020. The Partial Correlation Test (r) was used, controlled for confounding factors, to evaluate the correlation between the prevalence of overweight/obesity and the overall mortality rates due to COVID-19.

**Results:**

The mean mortality rate for COVID-19 in the period was 65.1 deaths per 100,000 inhabitants. Regarding the prevalence of obesity and overweight, 20.2% and 54.7% were the mean values observed in the state capitals, respectively. The prevalence of obesity was positively correlated with the overall mortality rate due to COVID-19, with mean positive correlation (r=0.380) and statistically significant correlation (p=0.034).

**Conclusion:**

This study pointed out that, at the aggregate level, there is a concomitant and correlated increase in mortality rates due to COVID-19 and prevalence of obesity in Brazilian capitals. The data found may contribute to actions to cope with the pandemic aimed at this population.

## INTRODUCTION

The first cases of pneumonia of unknown cause occurred at the end of 2019 in China (1). In January 2020, the etiological agent was identified and classified as Sars-CoV-2, a new species of Coronavirus (2) and, at the end of the same month, the World Health Organization (WHO) declared Coronavirus disease (COVID-19) a Public Health Emergency of International Importance (3). Brazil presented a mortality rate of 50.7 per 100,000 inhabitants until August 14, 2020, with important regional differences (4).

As research developed, more has been understood about the disease and its risk factors. COVID-19 was indicated to have worse prognosis and a higher risk of death when associated with obesity (5) and this condition has also been pointed out by several observational hospital-based studies (6-8) as an important factor associated with a worse prognosis for the infection. This association was also described for the SARS/MERS virus in previous publications, with indications of strong correlation between obesity and complications due to infection by other genetically similar to Sars-CoV-2 coronaviruses (5). Correlation with overweight condition was scarcely reported in the literature (6). To the day of this study, population-based research in the matter was also scarce.

Additionally, the situation in Brazil regarding obesity and overweight condition rates and the pandemic require special attention, due to the fact that the prevalence of obesity and overweight are high in the country and have increased significantly over the years (9). Accordingly, if these findings are extended to a populational level, the healthcare system will have another challenge in face of the pandemic: a significant and increasing portion of the population at higher risk of worse COVID-19 infection outcomes. Better understanding of the matter could be crucial to guide public health policies and avoid an even heavier healthcare system burden.

Considering the scarcity of population-based studies, the fact that hospital-based studies are susceptible to selection bias, that overweight condition and obesity rates are of high magnitude in Brazil and the possible relationship between these factors and higher mortality due to COVID-19, the necessity of a populational analysis becomes clear to better understand this relation at an aggregate level, helping to delineate health policies.

Therefore, our research aimed to evaluate the correlation between the prevalence of overweight condition and obesity with mortality rates due to COVID-19 in Brazil's state capitals at an aggregate level.

## MATERIALS AND METHODS

### Design and sample

This is an ecological study in which the units of analysis were the 26 state capitals of Brazil and the Federal District, aiming to investigate the existence of correlation of overweight and obesity prevalence, in adults (≥ 18), with general COVID-19 mortality rates. Data on prevalence, aggregate to capital level, for overweight condition, obesity and covariates were extracted from the *Vigilância de Fatores de Risco e Proteção para Doenças Crônicas por Inquérito Telefônico* 2019 (VIGITEL – Surveillance of Risk and Protective Factors for Chronic Diseases) survey (10). The general mortality rates due to COVID-19 were collected from the official website of the Brazilian Ministry of Health (MH) (4) and stratified by the same Brazilian capitals evaluated in the VIGITEL survey.

### Overweight and obesity data

Both overweight and obesity prevalence were extracted from the results of the *Vigilância de Fatores de Risco e Proteção para Doenças Crônicas por Inquérito Telefônico* 2019 (VIGITEL – Surveillance of Risk and Protective Factors for Chronic Diseases) survey. The VIGITEL is a population-based study which interviews adults (≥ 18) who live in residences with landline telephones in all 26 state capitals and the Federal District of Brazil and aims to understand this population's health in order to guide programs and actions that reduce the occurrence of chronic diseases. The VIGITEL's sampling methodology allows to compare capital cities risk factor and protection estimates, between adult inhabitants. Detailed information on the sampling and data collection process was previously described (10). The Body Mass Index (BMI) is the parameter used by VIGITEL to determine overweight condition and obesity cutoffs and is calculated by dividing weight in kilograms by the height in square meters – both weight and height are self-reported within the survey. An individual with a BMI ≥ 25 kg/m^2^ was considered to be overweight and individuals with BMI ≥ 30 kg/m^2^ were considered to have obesity. It is worth noting that overweight condition, within VIGITEL, is a broader measure which also includes obesity (BMI ≥ 30 kg/m^2^), whereas obesity is a narrower concept.

### COVID-19 mortality data

General mortality rates due to COVID-19, during the period between the 1st and 29th Epidemiological Week of 2020 (11), were collected from the official website of the Brazilian Ministry of Health (MH). Mortality rates in the website were calculated by multiplying the number of confirmed COVID-19 deaths of resident population – TCU's (*Tribunal de Contas da União*) 2019 estimated population for FPM's (*Fundo de Participação dos Municípios*) quotas (12) – by 100.000 (4). The process of updating data on cases and deaths confirmed by COVID-19 in Brazil was carried out daily by the Ministry of Health, through official information provided by the State Health Secretariats of the 27 Brazilian Federative Units (4). Data was stratified by Brazilian capital cities, but they are not discriminated by age, socioeconomic condition or sex.

### Covariates

The prevalence of diabetes mellitus (DM), systemic arterial hypertension (SAH) and smoking were selected as covariates, since they were previously described as risk factors for COVID-19 infection (13). All prevalence was obtained from the VIGITEL 2019 survey as well. We considered, in this study, the VIGITEL's estimated prevalence of diabetes mellitus and systemic arterial hypertension assessed by personal statement of whether a diagnosis was already established by a doctor, regardless of treatment. These variables were collected through the questions: “Has any doctor ever told you that you have diabetes?” and “Has any doctor ever told you that you have high blood pressure?”. The individual who answered positively to the question “Do you currently smoke?” was considered a smoker, regardless of number of cigarettes, frequency and duration of the smoking habit.

### Analysis

The data were represented by means with standard deviation (SD), medians, minimum and maximum values. The graphical representation was made using a scatter plot. The variables obesity, overweight and mortality due to COVID-19 were also stratified by Brazilian capitals. We used the Partial Correlation Test (r), controlled for confounding factors, to evaluate the correlation between the prevalence of overweight/obesity and the overall mortality rates due to COVID-19. The use of correlation tests between similar measures was previously described (14). This technique was chosen because it allows the evaluation of the pure relationship between two variables, after statistically eliminating the influence of other independent variables. Cohen's parameters (15) were used for the interpretation of correlation values (r): between 0.10 and 0.29 to indicate a non-existent or small correlation, between 0.30 and 0.49 to indicate that there is a mean correlation and between 0.50 and 1 to indicate a large correlation. Data was analyzed with the program IBM (SPSS^®^) version 25. The variables DM, SAH and smoking were used as control variables. The significance level adopted was 5% (p < 0.05).

### Ethical aspects

The VIGITEL was approved by the National Committee of Ethics in Research. (CAAE: 65610017.1.0000.0008). The mortality rates due to COVID-19 are available for public access, without the identification of participants and its use exempt approval of the Ethics Committee, according to resolution no. 510, of April 7, 2016, of the National Health Council.

## RESULTS

The mean mortality rate due to COVID-19 was 65.1 deaths per 100,000 inhabitants in Brazil's state capitals, with a minimum coefficient of 6.9/100,000 in Campo Grande (MS) and a maximum of 135.5/100,000 in Belém (PA). Regarding the prevalence of obesity and overweight, averages of 20.2% and 54.7% were observed in these state capitals, respectively (Table 1).

**Table 1:** Description of mortality rates, prevalence of obesity and overweight according to Brazilian capitals and description of the mean, median, minimum and maximum values of the study's covariates. Capital cities of Brazil

Brazilian capitals	COVID-19 mortality rate*	Obesity	Overweight
Aracaju	69.1	20.6	53.6
Belém	135.5	19.6	53.3
Belo Horizonte	13.6	19.9	52.5
Boa Vista	85.9	21.2	54.3
Campo Grande	6.9	22.5	58
Cuiabá	69.5	22.5	55.8
Curitiba	18.4	19.4	53.7
Florianópolis	7.6	17.8	53.6
Fortaleza	134.3	19.9	55.6
Goiânia	20.4	19.5	52.7
João Pessoa	64.0	20.4	54.7
Macapá	64.4	22.9	53.3
Maceió	65.9	20	54.4
Manaus	89.9	23.4	60.9
Natal	75.0	22.5	56.6
Palmas	9.4	15.4	49.9
Porto Alegre	13.7	21.6	59.2
Porto Velho	85.9	19.9	56.6
Recife	123.7	21.7	59.5
Rio Branco	75.9	23.3	56.6
Rio de Janeiro	114.6	21.7	57.1
Salvador	53.6	18.1	51.8
São Luís	96.1	17.2	50.3
São Paulo	71.6	19.9	55.8
Teresina	68.6	17.6	52.7
Vitória	88.7	17.6	49.1
Distrito Federal	36.0	19.6	55
**Mean (SD)**	**65.1 (38.8)**	**20.2 (2.0)**	**54.7 (2.8)**
Median	69.1	19.9	54.4
Minimum	6.9	15.4	49.1
Maximum	135.5	23.4	60.9
	**Diabetes Mellitus**	**Systemic Arterial Hypertension**	**Smoking**
Mean (SD)	6.8 (1.1)	23.3 (3.4)	8.3 (2.7)
Median	6.8	24.3	7.9
Minimum	4.6	16.9	4.4
Maximum	8.6	28.5	14.6

Weighted prevalence (VIGITEL. 2019).

* Estimated for every 100.000 inhabitants.

**Figure 1:**
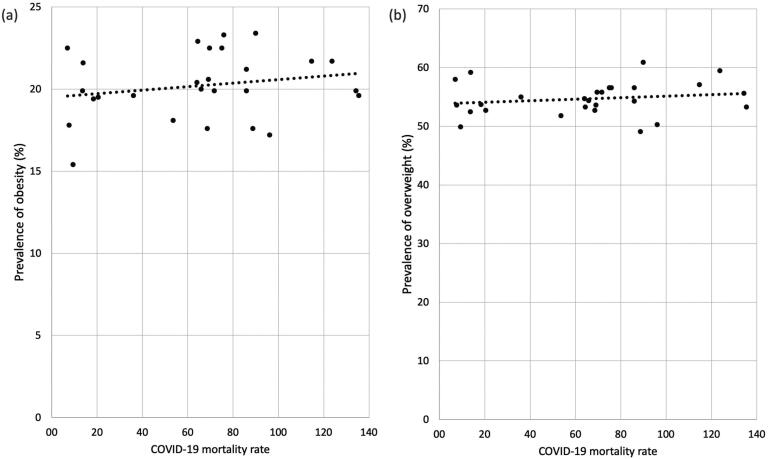
(**A**) Scatter plot between prevalence of obesity and mortality rates by COVID-19; (**B**) Scatter plot between prevalence of overweight and mortality rates by COVID-19. Capital cities of Brazil.

Figure 1 shows the dispersion graph between the prevalence of obesity (a), overweight (b) and the mortality rates due to COVID-19 in Brazilian capitals. Visually, a greater slope of the line is observed between the prevalence of obesity and mortality due to COVID-19.

The prevalence of obesity was positively correlated with the overall mortality rate due to COVID-19, with statistically significant (p=0.034) and mean positive correlation (r=0.380). Although no statistical significance was evidenced, we also observed a mean positive correlation (r=0.367) between COVID-19 mortality rate and overweight (Table 2).

**Table 2:** Correlation between overweight and obesity (2019) and mortality rates due to COVID-19 (2020) in the capital cities of Brazil

Variables	COVID-19 mortality rate	
	r*	p-value
Overweight	0.367	0.078
Obesity	0.380	0.034

r: Partial Correlation coefficient.

* Correlation adjusted for diabetes, hypertension and smoking variables.

## DISCUSSION

Corroborating our findings, prospective and hospital-based articles found an association between obesity and progression to the severe form of COVID-19 infection (6,7), obesity as a risk factor for mortality (8,16) and longer hospital stay for these patients (17). Moreover, Palaiodimos and cols. (18) reported that severe obesity is independently associated with a worse prognosis and mortality. Although the underlying mechanisms are still unclear, literature has indicated potential reasons why obesity may be a risk factor for severe COVID-19 infection and higher mortality: the presence of uncontrolled chronic obesity-related comorbidities (19), impaired pulmonary function, elevated angiotensin-converting enzyme 2 (ACE2) expression (20), chronic inflammation (13), oxidative stress and lipotoxicity (5). Cardiac damage, aggravated inflammatory response and increased coagulation activity were correlated to mortality amongst patients with obesity (16). Furthermore, evidence has shown that obesity has been linked to increased susceptibility to infections in general (5).

Regarding overweight condition, this study did not verify a population-level correlation between overweight and mortality due to COVID-19 and literature on the matter is scarce (6). In Brazil general overweight and obesity rates have increased with time and the situation is challenging. The country has a national strategic plan that aims to combat non transmissible chronic diseases (*Plano de Ações Estratégicas para o Enfrentamento de Doenças Crônicas Não Transmissíveis*) (21), and one of its purposes is to halt the growth of both overweight and obesity rates. The VIGITEL survey – which supplies the aforementioned plan – shows that, from 2006 to 2019, overweight and obesity rates increased from 42.6% to 55.4% and 11.8% to 20.3%, respectively (10). In face of a pandemic this data is particularly concerning because of the possible impacts to the National Healthcare System (SUS – *Sistema Único de Saúde*) in terms of occupation, expenditure and availability – since an expressive portion of brazilian population may be at higher risk of worse prognosis. Greater understanding of how COVID-19 may impact people with overweight condition and obesity at a populational level – the aim of this study – is of great importance to delineate Healthcare strategies and public policies, preventing and preparing for potential worsened scenarios.

Limitations of this study must be considered when interpreting the results. Two secondary data sources were included in this study, one (VIGITEL) is a population-based survey and the other is a mortality rate information system (Brazilian Ministry of Health (MH) website). Vigitel (10) is based on the interviewees' report, which may have led to information bias. However, researchers attest to the validity of this strategy, demonstrating high sensitivity (> 91%) and specificity (> 83%) values for BMI, calculated via self-reported height and weight (22). Also, the source of information used to verify deaths due to COVID-19 is influenced by the testing capacity of the capital cities observed, as well as by the ability to notify and monitor epidemiological surveillance of each site, with recognized underreporting of cases and deaths (23) and important regional differences (4). It is important to highlight though that the mortality rates information system in Brazil evolves in terms of quality and coverage, generating reliable data for research (24).

In addition, an important limitation concerns variables such as age, socioeconomic condition and sex. The prevalence measures used here refer to resident adults aged 18 years or older, whereas the mortality rate refers to all resident individuals, since the database does not discriminate rates by age. Therefore, although deaths due to COVID-19 mostly occur among adults (25), this fact may have influenced the observed results. Equally important, socioeconomic condition and sex variables couldn't be evaluated due to the same database restrictions. In order to evaluate these factors, other sources would have to be included in the analysis, compromising comparability and interfering in the results' heterogeneity. Therefore, VIGITEL and the HM website were added as the only sources.

Moreover, the epidemiological design itself is susceptible to aggregation bias or ecological fallacy, therefore, the relationship observed between variables at the aggregate level may not be valid at the individual level – although, as mentioned before, hospital-based prospective studies results were consistent with our findings.

We were able to conduct a population based study that, to the best of our knowledge, is the first investigation addressing the relationship between prevalence of overweight condition and obesity with mortality rates due to COVID-19 nationwide. In conclusion, our results showed that, at aggregate level, there is a concomitant and correlated increase in mortality rates due to COVID-19 and prevalence of obesity in Brazilian capitals. Additional studies are needed to confirm and expand our results. Better understanding in this matter, especially at a population level, is crucial in order to support focused public policies.
